# Trifluridine/Tipiracil: Old Drug, New Tricks

**DOI:** 10.6004/jadpro.2016.7.4.7

**Published:** 2016-05-01

**Authors:** Kate D. Jeffers

**Affiliations:** UCHealth–Memorial Hospital, Hematology/Oncology, Colorado Springs, Colorado

Colorectal cancer is the third most commonly diagnosed cancer in the United States, with an estimated 134,490 new cases in 2016. Colorectal cancer is the third leading cause of cancer death in both men and women in the United States, with an estimated 49,190 deaths expected to occur in 2016 (American Cancer Society [ACS], 2016b).

Colorectal cancer develops in the colon or the rectum, usually over a period of 10 to 20 years. Most colorectal cancers begin as noncancerous polyps (or adenomas) and may progress into cancerous masses as the polyp grows. Approximately 96% of colorectal cancers are classified as adenocarcinomas.

Risk of developing colorectal cancer increases with age, with 90% of cases occurring after the age of 50 (ACS, 2014). Risk factors for developing colorectal cancer include age, male gender, inflammatory bowel disease, personal or family history of polyps, genetic syndromes such as familial adenomatous polyposis (FAP) or hereditary nonpolyposis colorectal cancer (Lynch syndrome), and lifestyle factors. Lifestyle factors associated with an increased risk of colorectal cancer include smoking, consumption of red and processed meats, alcohol consumption, diabetes mellitus, low levels of physical activity, obesity or high body mass index, and metabolic syndrome (National Comprehensive Cancer Network [NCCN], 2016).

Colorectal cancer is staged with regard to bowel wall and muscle invasion, lymph node involvement, and distant metastatic spread. Staging is usually performed following surgical exploration and removal, if possible. The 5-year survival rates decrease with increasing stage. For colon cancer, stage 1 disease has a relative 5-year survival rate of 92%, whereas stage IV disease has a relative 5-year survival rate of approximately 11%. The survival rates for rectal cancer differ slightly; stage 1 disease has a relative 5-year survival rate of 87%, and stage IV disease has a relative 5-year survival rate of approximately 12% (ACS, 2016).

Fluoropyrimidines have long been the cornerstone of chemotherapy treatment for stages II–IV colorectal cancer, with adjuvant chemotherapy being considered in patients with high-risk stage II disease and recommended in patients with stage III disease (NCCN, 2016). Fluoropyrimidines, including fluorouracil and capecitabine, act primarily as inhibitors of thymidylate synthase (TS), leading to cytotoxic nucleotides and cell death (DeVita, Lawrence, Rosenberg, DePinho, & Weinberg, 2011). Fluorouracil may also be combined with leucovorin (folinic acid) to enhance the TS binding capacity. In advanced or metastatic disease, initial treatment regimens often combine these medications with oxaliplatin (e.g., FOLFOX or CapeOx) or irinotecan (FOLFIRI) with or without a vascular endothelial growth factor inhibitor (e.g., bevacizumab [Avastin] or ramucirumab [Cyramza]) or an epidermal growth factor inhibitor (e.g., cetuximab [Erbitux] or panitumumab [Vectibix]).

Despite appropriate initial treatment, many patients will progress. Subsequent lines of therapy may include different combinations or single agents of the medications listed here, regorafenib [Stivarga], or the newly approved agent trifluridine/tipiracil (Lonsurf; TAS-102; NCCN, 2016).

TAS-102 is an orally administered combination of a thymidine-based nucleoside analogue, trifluridine, and a thymidine phosphorylase inhibitor, tipiracil hydrochloride (Taiho Oncology, 2015). TAS-102 was approved on September 22, 2015 (US Food and Drug Administration [FDA], 2015) and is indicated for the treatment of patients with metastatic colorectal cancer who have been previously treated with fluoropyrimidine-, oxaliplatin-, and irinotecan-based chemotherapy, an anti-VEGF (vascular endothelial growth factor) biologic therapy, and if RAS wild-type, an anti-EGFR (epidermal growth factor receptor) therapy (Taiho Oncology, 2015).

## PHARMACOLOGY

Trifluridine was originally synthesized by Heidelberger and colleagues in 1964 (Hong et al., 2006). Trifluridine is an analogue of thymidine with two mechanisms of antitumor action. First, it inhibits TS, which is similar to fluorouracil. Second, it creates double-strand DNA breaks by incorporating the triphosphate form into DNA (Doi et al., 2012). Initial studies with intravenous trifluridine revealed a very short serum half-life of approximately 12 minutes with limited, short-lived disease responses. When administered orally, trifluridine is rapidly degraded to an inactive form by the enzyme thymidine phosphorylase (Hong et al., 2006).

Tipiracil is a thymidine phosphorylase inhibitor, which when administered with trifluridine, results in an improvement in the pharmacokinetic profile of trifluridine, allowing for increased drug concentrations and antitumor activity. TAS-102 consists of trifluridine and tipiracil combined at a molar ratio of 1:0.5 (Doi et al., 2012). Dosing is based on the trifluridine component. Preclinical xenograft studies in mice have revealed antitumor activity in cell lines resistant to fluorouracil (Mayer et al., 2015).

## CLINICAL TRIALS

Phase I clinical trials explored a number of dosing strategies in various tumor types. Dosing schedules included 50 mg/m²/day of the trifluridine component once daily on days 1–14 every 21 days (Hong et al., 2006), 80 mg/m²/day of the trifluridine component divided three times daily on days 1–5 and 8–12 every 28 days (Overman et al., 2008), 100 mg/m²/day of the trifluridine component once daily on days 1–5 and 8–12 every 28 days, 160 mg/m²/day of the trifluridine component once daily on days 1–5 every 21 days (Overman et al., 2008), and the FDA-approved dosing schedule of 70 mg/m²/day of the trifluridine component divided twice daily on days 1–5 and 8–12 every 28 days (Bendell et al., 2015; Doi et al., 2012).

The FDA-approved dosing schedule was further investigated in a double-blind, randomized, placebo-controlled, phase II trial involving 112 Japanese patients (intention-to-treat population) with metastatic colorectal cancer refractory to or intolerant of fluorouracil, irinotecan, and oxaliplatin. After a median follow-up of 11.3 months, the median overall survival was 9.0 months in the TAS-102 group and 6.6 months in the placebo group (hazard ratio for death, 0.56; *p* = .0011). No treatment-related deaths occurred. The side-effect profile was manageable, with 50% developing grade 3 or 4 neutropenia, 28% developing grade 3 or 4 leukopenia, and 5% developing grade 3 or 4 anemia (Yoshino et al., 2012).

The results of the phase I and II studies led to the phase III RECOURSE study. In this randomized, double-blind, multinational study, 800 patients with metastatic, refractory colorectal cancer were assigned to receive TAS-102 or placebo in a 2:1 fashion. For the purposes of this study, refractory was defined as patients who had received at least two prior regimens of standard chemotherapies, which could have included adjuvant chemotherapy if a tumor had recurred within 6 months after the last treatment; if they had tumor progression within 3 months after the last treatment; or if clinically significant adverse events precluded the readministration of standard therapies. Study treatment was administered as TAS-102 or placebo at 70 mg/m²/dose of the trifluridine component divided twice daily, after morning and evening meals, on days 1–5 and 8–12 every 28 days.

The primary endpoint was overall survival. Secondary endpoints included progression-free survival, response rate, rate of disease control, and safety. Baseline demographic and disease characteristics were well balanced between the study groups.

The median overall survival was 7.1 months for patients in the TAS-102 group and 5.3 months for patients in the placebo group (hazard ratio for death, 0.68; *p* < .001). The median progression-free survival was 2.0 months in the TAS-102 group and 1.7 months in the placebo group (hazard ratio for progression, 0.48; *p* < .001). The overall survival and progression-free survival benefit for TAS-102 was observed in all prespecified subgroups. Objective response rates were 1.6% with TAS-102 and 0.4% with placebo (*p* = .29); disease control was 44% in the TAS-102 group and 16% in the placebo group (*p* < .001; Mayer et al., 2015).

## ADVERSE EFFECTS

In the RECOURSE study, grade 3 or higher adverse events occurred more frequently in the group receiving TAS-102 (69%). The most common laboratory abnormalities of grade 3 or higher included neutropenia (38%), leukopenia (21%), anemia (18%), and thrombocytopenia (5%). Febrile neutropenia was reported in 4% of patients receiving TAS-102 compared with no patients receiving placebo; 9% of patients who developed neutropenia received granulocyte colony-stimulating factor, with one treatment-related death resulting from septic shock. Additional laboratory abnormalities of grade 3 or higher in the TAS-102 arm were similar to those in the placebo group: increased alanine aminotransferase levels (2% vs. 4%), increased aspartate aminotransferase levels (4% vs. 6%), increased total bilirubin (9% vs. 12%), increased alkaline phosphatase levels (8% vs. 11%), and increased serum creatinine (< 1% in both groups).

In addition to laboratory abnormalities, patients in the TAS-102 group were more likely to experience side effects similar to those of other fluoropyrimidine treatments. Nausea of any grade occurred in 48% of patients (2% grade 3 or higher), decreased appetite in 39% (4% grade 3 or higher), diarrhea in 32% (3% grade 3 or higher), fatigue in 35% (4% grade 3 or higher), stomatitis in 8% (< 1% grade 3 or higher), and hand-foot syndrome in 2% (no grade 3 or higher; Mayer et al., 2015).

## DOSING AND TOXICITY MANAGEMENT

TAS-102 is available in 2 tablet sizes: 15 mg of trifluridine/6.14 mg of tipiracil and 20 mg of trifluridine/8.19 mg of tipiracil. The recommended starting dose is 35 mg/m² (rounded to the nearest 5 mg of the trifluridine component), taken twice daily on days 1–5 and 8–12 of a 28-day cycle. The maximum single dose based on the trifluridine component is 80 mg. Cycles are continued until disease progression or unacceptable toxicity (Taiho Oncology, 2015).

In the RECOURSE study, a total of 73 (14%) patients required dose reductions, with 53 (10%) patients having a single dose reduction, 18 (3%) having two dose reductions, and 2 (< 1%) having three dose reductions (Mayer et al., 2015). Complete blood cell count with cell differential should be performed prior to each cycle and on day 15 of the cycle. Dose modifications or therapy delays are recommended for neutropenia, thrombocytopenia, and other grade 3 or 4 nonhematologic toxicities (Table). After count recovery, TAS-102 may be resumed at a 5 mg/m² reduction from the previous dose level. A maximum of 3 dose reductions may occur to a minimum dose of 20 mg/m² twice daily. Dose escalation should not be performed (Taiho Oncology, 2015).

**Table:**
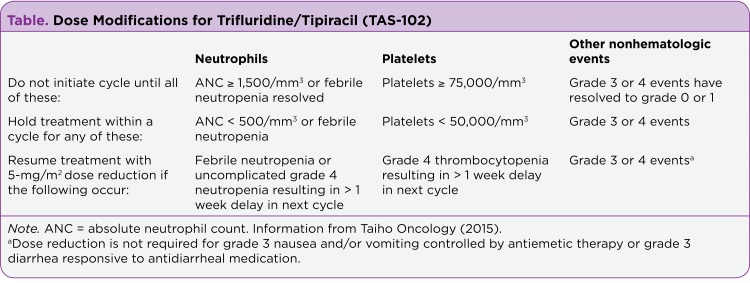
Dose Modifications for Trifluridine/Tipiracil (TAS-102)

In patients administered a standard high-fat, high-calorie meal, a decrease in trifluridine C_max_ (maximum concentration) was observed. Increased trifluridine C_max_ has an observed correlation with decreased neutrophil counts. Therefore, to decrease the risk or severity of neutropenia, TAS-102 should be taken within 1 hour of the morning and evening meals (Taiho Oncology, 2015) with a full glass of water. These dietary restrictions were employed within clinical trials (Mayer et al., 2015).

Trifluridine and tipiracil are not metabolized by cytochrome P450 (CYP) enzymes. In vitro studies have not shown any inhibitory or inductive effects on CYP1A2, CYP2B6, or CYP3A4/5. Additionally, TAS-102 has not been shown to cause QTc prolongation (Taiho Oncology, 2015). No clinically significant drug interactions have been identified at this point.

## PATIENT COUNSELING

Advanced practitioners are well positioned and well educated to manage the side effects of chemotherapy medications. As with other oral cancer therapies, patient education on side effects and management strategies prior to starting therapy is essential. Education should include emphasis on the proper administration techniques, including tablet strength if the patient will receive both dosage strengths; the dosing schedule; and safe handling instructions. TAS-102 should be taken within 1 hour of the morning and evening meals. The importance of compliance with laboratory draws should be emphasized. At therapy initiation, patients should be made aware of the risk for neutropenia, thrombocytopenia, and anemia, as well as management strategies and precautions to take at home. Emergency contact numbers and instructions should be provided to all patients in the event of symptoms of an infection or other side effects.

Gastrointestinal side effects, such as nausea, vomiting, and diarrhea, should be explained to the patient. Antinausea medications, such as a 5-HT3 (5-hydroxytryptamine) antagonist or other therapy in accordance with guideline recommendations, may be provided to the patient in advance. TAS-102 is not currently included in the NCCN Guidelines (NCCN, 2015). Antidiarrheal medications may be recommended to the patient to include the use of loperamide. Patients should be advised to contact their provider for severe or persistent nausea/vomiting or diarrhea not relieved with over-the-counter treatments. Patients should be advised to report any other side effects to their provider for management.

Lastly, patients should be advised to contact their provider before the addition of any herbal or natural supplement, vitamins, or over-the-counter medications. Although clinically significant drug interactions have not been reported with TAS-102, an accurate medication list should be maintained at all times for patients receiving chemotherapeutic agents.

## SUMMARY

Trifluridine/tipiracil is a new and valuable addition for the treatment of colorectal cancer, indicated for patients with metastatic colorectal cancer who have been previously treated with fluoropyrimidine-, oxaliplatin-, and irinotecan-based chemotherapy, an anti-VEGF biologic therapy, and if RAS wild-type, an anti-EGFR therapy. Like other oral cancer therapies, patient education, adherence, and toxicity monitoring are required. Patients should confirm understanding of the dosing schedule prior to initiating treatment. Laboratory parameters should be drawn every 2 weeks during therapy, with appropriate dose adjustment for neutropenia or thrombocytopenia. Advanced practitioners are ideally positioned to facilitate appropriate follow-up and toxicity management for patients receiving trifluridine/tipiracil.
